# Training in the prevention of cervical cancer: advantages of e-learning

**DOI:** 10.3332/ecancer.2015.580

**Published:** 2015-10-08

**Authors:** Assumpta Company, Mireia Montserrat, Francesc X Bosch, Silvia de Sanjosé

**Affiliations:** 1 e_Oncología Cancer Epidemiology Research Programme (PREC) Catalan Institute of Oncology (ICO), Barcelona, Spain; 2 Consortium of Biomedical Research in Epidemiology and Public Health (CIBERESP), Barcelona, Spain; § Cancer Epidemiology Research Programme, e-oncology, Catalan Institute of Oncology, Av. Gran Via de l’Hospitalet 199–203, 08908 L’Hospitalet de Llobregat, Spain

**Keywords:** distance education, cervical cancer, HPV, prevention, early diagnosis, vaccines, screening

## Abstract

Cervical cancer remains the second most common cancer for women worldwide and is the cancer priority in most low- and middle-income countries (LMIC). The development of vaccines against the human papilloma virus (HPV) and the impact of technology both for the detection of HPV and cervical cancer represent milestones and new opportunities in prevention. New internet-based technologies are generating mass access to training programmes. This article presents the methodology for developing an online training programme for the prevention of cervical cancer as well as the results obtained during the four year period wherein the same programme was delivered in Latin America.

## Background

Cervical cancer remains the second most common cancer for women worldwide and is one of the cancer priorities in most LMIC [1].

Unlike other types of cancer, cervical cancer is a disease that is preventable and curable if it is diagnosed and treated at an early stage. However, the absence of an effective prevention strategy leads to a late diagnosis and converts the disease into one of the leading causes of death in young women. The result is that half a million new cases of cervical cancer are diagnosed every year worldwide, and nearly a quarter of a million women die of this disease [2, 3].

The development of vaccines against HPV and the impact of HPV technology for the detection of cervical cancer represent milestones and new opportunities for prevention.

Information on new vaccines and new screening options must be scientifically accurate and technically impartial. The information must be accurate, informative, and disseminated honestly. The introduction of a new prevention technology worldwide means that tens of thousands of health professionals and managers need to understand its significance and use, and they in turn must be able to transmit this information appropriately to millions of families living in environments with completely different languages, cultures, and beliefs.

Health professionals often do not have many opportunities to update their knowledge and skills, and the same is true of other professionals such as nurses and public health professionals [4, 5].

New internet-based technologies have provided unique possibilities for medical training without the barriers of distance, time, and space. They also facilitate widespread access to and consultation with the best cancer specialists from around the world [6].

This is the context in which the Catalan Institute of Oncology’s (ICO) Cancer Epidemiology Research Programme has been working on for the last 10 years, developing an extensive training and information programme on HPV and associated diseases. This programme is aimed at a broad spectrum of professionals: from specialists in the area who are required to keep abreast of the latest developments to professionals who require basic knowledge for their daily clinical practice and health planners.

## Methodology

The course presented here is part of ICO’s worldwide training programme on HPV and the prevention of associated diseases.

The main components of ICO’s education programme in the field of cervical cancer are:

1. The World Health Organisation (WHO)/ICO Information Centre on HPV and cancer (www.hpvcentre.net/index.php).
2. ICO’s International Monographs Programme on HPV and the prevention of associated diseases.
3. Online course on the prevention of cervical cancer.

The WHO/ICO Information Centre on HPV and cancer: (www.hpvcentre.net/index.php)

The ICO in cooperation with the WHO launched this initiative in 2004. The mission of the Information Centre is to collect, edit, and spread scientific information on the HPV virus and associated diseases worldwide through the web page, completely free of charge.

ICO’s International Monographs Programme on HPV and the prevention of associated diseases.

With respect to the Information Centre and thanks to the collaboration of the international scientific community on HPV, a number of international scientific reviews have been published in scientific papers and regional reports. In 2006, the first monograph in the series was published and since then ten regional reports have been published. In 2012, an update of the general report was published.

In preparing the monographs more than 500 international experts participated as editors, authors, or reviewers. The scientific content of the monographs has been the basis for developing the virtual course that is presented here.

Online course on the prevention of cervical cancer

In order to maximise the impact of the revisions included in the monograph programme, in 2011 an 18-hour online course on the prevention of cervical cancer was offered on the e-oncologia virtual platform.

The course has been developed jointly with the International Federation of Gynaecology and Obstetrics (FIGO), the Union for International Cancer Control (UICC), the International Atomic Energy Agency (IAEA), the International Agency for Research on Cancer (IARC), and the World Health Organisation (WHO).

**Figure 1.**
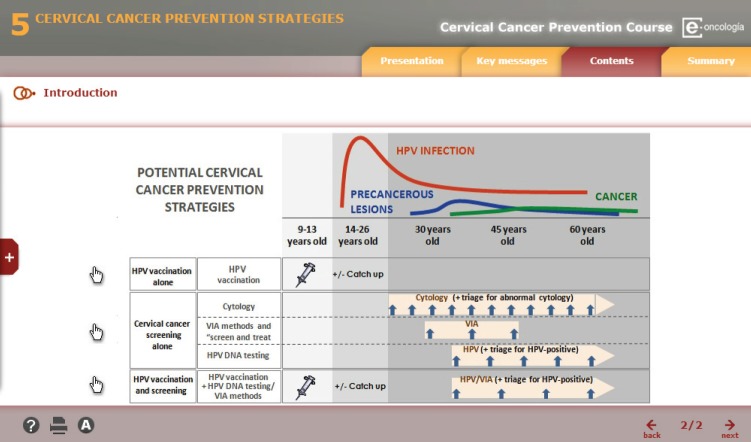
Example of digital content.

The methodology for the preparation of course content was carried out using the following stages:

- Selection of the most significant material included in the ICO monographs.
- Adapting the contents to a format suitable for distance learning based on the guidelines and templates established by e-oncologia.
- A two-day peer review meeting was held in Barcelona in January of 2011 in order to review and adapt the content. A total of 15 professionals participated including representatives from each of the participating institutions, independent experts of recognised prestige in the area, and the authors of the content. At the meeting the authors presented the course content and after discussion with the rest of participants, a consensus was reached on the final content of the course.
- Paedagogical treatment and digitisation of content in e-learning format was carried out for training using a virtual platform. The digital content of the course had been developed using interactive elements and multimedia to facilitate learning (Figure 1).
- Training of tutors in the use of digital tools and on-line training.

The final course table of contents:

- Module 1. Introduction
- Module 2. Natural history of HPV infections and cervical cancer
- Module 3. Burden of HPV infections and cervical cancer
- Module 4. Other HPV-associated diseases.
- Module 5. Control and prevention of cervical cancer:
  - 5.1. HPV vaccine.
  - 5.2. Male condom and male circumcision.
  - 5.3. Current options for cervical cancer screening.
  - 5.4. Strategies for the prevention of cervical cancer.
  - 5.5. Education and health guidance.
- Module 6. HPV in specific populations.
- Module 7. Visual inspection with acetic acid test (VIA)

Duration: 18 hours of training to be completed in four weeks.

Students can complete all the modules or only those that are of interest to them because of their specialty, for example vaccines in the case of paediatricians.

**Figure 2.**
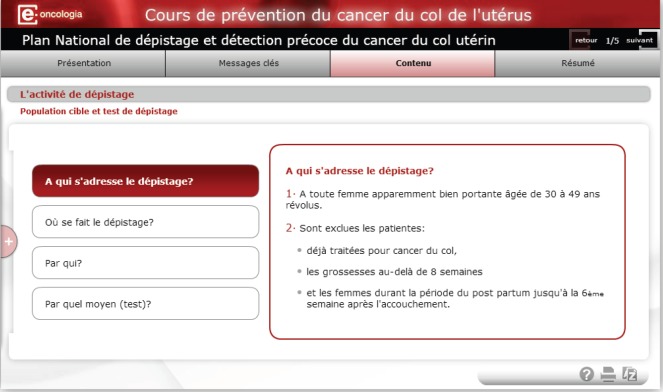
Module on Morocco’s national screening plan.

**Figure 3.**
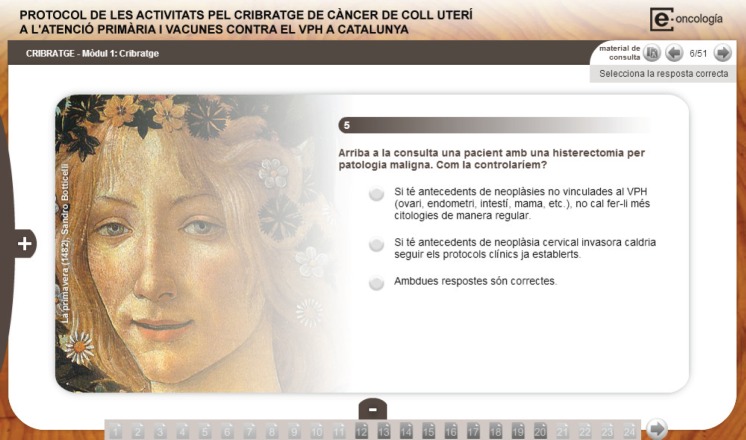
Module on Catalonia’s screening protocol.

The modular structure of the course allows for the addition of specific modules including the prevention and screening protocols for the country or region of interest. In Figures 2 and 3, examples are included of specific modules on Morocco’s National Cancer Prevention and Control Plan (NCPCP) and on Catalonia’s Screening Protocol based on the resolution of clinical cases.

The course is aimed at a wide range of professionals including:

- Health professionals involved in the prevention of cervical cancer, gynaecologists, paediatricians, microbiologists, primary care physicians, and vaccination technicians.
- Public health professionals, health managers and planners, researchers and educators.
- Specialist nurses and midwives.

The evaluation model has been based on e-oncology standards. Academic outcome, continuity, and satisfaction indicators will be monitored.

In evaluating the programme the following indicators are taken into account:

- General usage statistics (internal campus reports), access, study time, and drop-out rate before starting and during the course. The drop-out rate prior to the start is an indicator of the suitability of participant selection. The dropout rate during the course is a less specific indicator since it is estimated from the number of students who do not take the final exam and therefore includes different student profiles. These include students that have not completed the course because of lack of satisfaction; students that have not completed because they are only interested in some of the modules, such as vaccines, and students who having completed the course do not take the final exam because they are not interested in accreditation.
- Percentage of students who successfully completed and passed the course and therefore obtained the accreditation.
- Overall satisfaction on the basis of a quality questionnaire that students complete at the end of the course. The questionnaire must be filled out in order to download the accreditation certificate, and so the majority of replies come from students who have completed the course and taken the final exam (quality questionnaire).

For the individual assessment of each student, a test of knowledge is taken. This is a test-type evaluation consisting of 30 questions. To pass the test, the student must correctly answer 70% of the questions. The student has two opportunities to answer the exam questions. For each new attempt the system modifies the order in which questions are presented, and it randomly selects 30 from a pool of 50 options. The test is automatically corrected by the system (Learning management system or LMS).

Once the student passes the knowledge test, he/she receives CME accreditation from the Accreditation Council of Oncology in Europe (ACOE); CME recognition from the American Medical Association (AMA); a special accreditation granted by the Capacity Building in Education and Training Committee (CBETC) of the International Federation of Gynaecology and Obstetrics (FIGO).

For course dissemination a *‘cascade’* expansion model has been used in which the best students can become tutors for future course presentations, and use the course to train groups of students in their area of influence: institutions, scientific associations, etc. In addition to allowing for the optimised dissemination of the course, this model also ensures that the tutoring is provided by professionals who are knowledgeable about local and regional realities.

## Results

There are two modes of access to the course: classrooms that are permanently open with free access to any participant and closed or premium classes for specific groups of students with similar interests selected by the tutor.

The open classroom model is similar to the Massive Open Online Course (MOOC) platforms, i.e. it is accessible to a large number of participants without cost. Unlike the standard MOOC model, in our case registered students who have not logged-in receive a weekly follow-up e-mail, and students can consult tutors who are permanently available with their technical and scientific questions.

In the case of the premium classroom, this is the classic virtual training model, i.e. a specific number of students are invited to take the course, for a specific amount of time during which they have a tutor at their disposal usually from their own institution or region.

Below we offer the results obtained worldwide for both the open classroom and the premium. Further on we will carry out a more detailed analysis specifically for Latin American students, differentiating the two models of delivery.

**Figure 4.**
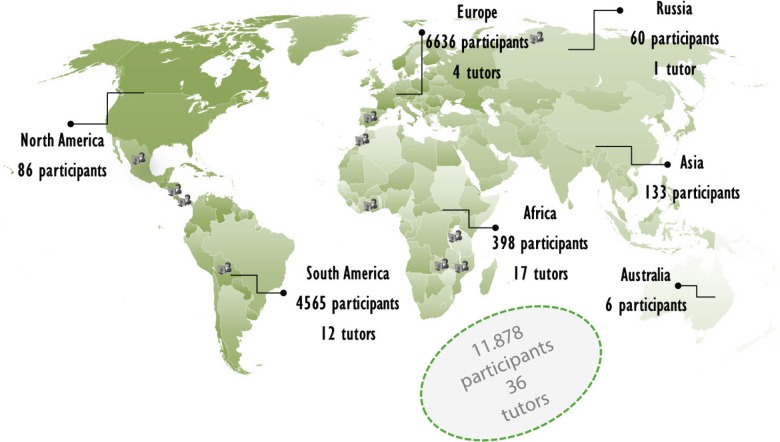
Distribution of students by continent.

Figure 4 shows the distribution of enrolled students and tutors by continent (2011–2014)

As can be seen in Figure 4, the majority of participants come from Spain and Latin America.

Table 1 shows the distribution of students according to language.

Globally, 70% of enrolled students completed the course and got their diploma.

Table 2 shows the distribution of all Latin American students according to country of origin.

Academic results—open classroom

In the case of the open classroom, since all participants access the classroom at least once at the time of registration, the data that we can use to evaluate both the academic result and the dropout rate is the number of students who take the final examination. The rest are counted as having dropped out during the course. In Table 3, we see the results grouped for all participants of the course given in Spanish.

The completion rate is very good when compared with the data obtained from the majority of MOOC courses in which the completion rate ranges from 6–29% only [7]. When analysing the results by country, for countries with more than 100 students, we observed a decrease in success rate but always well above the results usually seen in virtual activities given in this modality (Table 4).

Academic results—premium classrooms

In Latin America, premium classrooms have been established in Mexico and Colombia. In all the countries analysed, the results obtained for students participating in this modality are better than for those using the open classroom (Table 5). The data is shown in Tables 6 and 7.

Since 2012, a total of 924 students from Mexico had taken the course in a premium classroom with a tutor from Mexico. As can be seen in Table 6, the success rates are, except in one case, above 70%.

In the case of Colombia, almost 600 students had participated in the premium classroom since 2012. The results are detailed in Table 7 and are very similar to those of Mexico although with somewhat higher drop-out rates at the beginning.

**Figure 5.**
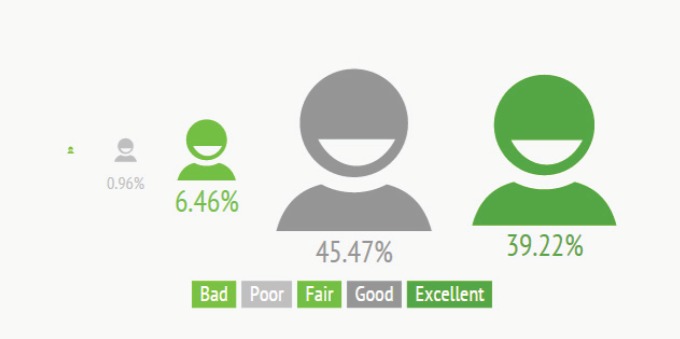
Overall results of the satisfaction survey.

Satisfaction survey results:

At the end of the course, students must answer a questionnaire to assess their degree of satisfaction. Eighty-five percent (85%) of participants rated the course as good or excellent. The results were very similar across all countries and delivery modalities, since as has been mentioned above, the questionnaire is only compulsory for those students who have finished the course and want to download their certificate. This is probably the reason why the profile is very homogeneous (Figure 5).

## Conclusion

As has been shown in the **e-oncología** programme on the prevention of cervical cancer, e-learning has demonstrated its utility in continuing education for health professionals regardless of their place of work or residence. The analysis of the results indicates that:

- Even with a MOOC type model of delivery it is important to monitor and tutor students to ensure high success rates.
- The most optimal model is one which brings together groups with similar interests, in which the scientific tutoring is given by professionals of the same institution, and/or region.
- Course content should be adapted and adjusted to different national realities.
- Ongoing tutor support is necessary for prior content and virtual skills training, as well as throughout course delivery.
- Independence in the management and publication of content is beneficial.

The main achievements that have been made are:

- The incorporation into a knowledge network of the best national, regional, and international reference persons for each disease, theme, and specialty as authors and tutors.
- A consolidated paedagogical model which is constantly being innovated.
- The accreditation of universities and scientific societies of recognised national and international prestige.

This same model can be extended and applied to any of the other oncology specialties in Spain and Latin America.

## Conflict of interest

Student’s grants have been provided by: GlaxoSmithKline, Merck & Co and Sanofi Pasteur MSD. None of the sources of funding had any participation directly or indirectly in the preparation of the materials, selection of authors and tutors, topics or references.

## Figures and Tables

**Table 1.** Distribution of students by language.

Language	Students	%
**Spanish**	10,990	92.90%
**English**	574	4.85%
**French**	266	2.25%
**Russian**	48	0.4%
**Total**	**11,878**	

**Table 2.** Origin of students by country (countries with more than 100 students).

**Mexico**	1,640	35.93%
**Peru**	1,125	24.60%
**Colombia**	761	16.67%
**Argentina**	137	3.00%
**Ecuador**	104	2.30%
**Paraguay**	104	2.30%
**Other**	694	15.20%
**Total**	**4,565**	

**Table 3.** Open classroom academic results for course in Spanish.

Registered Students	Students having completed the course	Drop-out rate	Completion rate
8,227	6,428	22%	78%

**Table 4.** Open classroom academic results by country^*^.

Country	Registered Students	Students having completed the course	Drop-out rate	Completion rate
**Mexico**	716	471	34%	66%
**Peru**	285	167	41%	59%
**Colombia**	178	105	41%	59%

* countries with more than 100 students registered.

**Table 5.** Definition of the indicators used to evaluate the academic results.

***Registered Students***	*All participants who have been invited to participate in the course.*
***Active students***	*All participants who have accessed a virtual classroom at least once.*
***Students having completed the course***	*Those that have taken and passed the final evaluation.*
***Beginning drop-out rate***	*Percentage of registered students that have never accessed the virtual classroom.*
***In course drop-out rate***	*Percentage of active students that do not take or quit the final exam.*
***Success rate***	*Percentage of active students that take and pass the final exam.*

**Table 6.** Academic results—premium classrooms in Mexico.

	Registered	Active	Completed	Beginning drop-out rate	Ongoing drop-out rate	Success rate
**June–July 2012**	45	23	19	48.89%	17.39%	82.60%
**October–November 2012**	218	170	167	22.02%	1.76%	98.23%
**April–June 2013**	135	131	75	2.96%	42.75%	57.25%
**October 8–November 2013**	142	132	129	7.04%	2.27%	97.72%
**October–November 2014**	238	233	216	2.10%	7.30%	92.70%
**February–March 2015**	146	129	102	11.64%	20.93%	79.06%

**Table 7.** Academic results—premium classrooms in Colombia.

	Registered	Active	Completed	Beginning drop-out rate	Ongoing drop-out rate	Success rate
**January–February 2012**	51	43	43	15.69%	0.00%	100%
**April–June 2012**	86	48	48	44.19%	0.00%	100%
**April–June 2012**	61	44	44	27.87%	0.00%	100%
**October–November 2013**	306	210	143	31.37%	31.90%	68.09%
**March–April 2014**	79	49	38	37.97%	22.45%	77.55%
